# Supplementary material for: A Comparison of Aerodynamic Parameters in Two Subspecies of the American Barn Owl (Tyto furcata)
Source: Animals (Basel). 2022 Sep 22;12(19):2532. doi: 10.3390/ani12192532 (PMC9558544; doi:10.3390/ani12192532)
Supplement: Supplementary file 1 [file animals-12-02532-s001.zip › animals-1900484-Table_S1_bodymass.pdf]

Table S1. Body-mass measurements of Galapagos barn owls

Number	Code of bird	Sex	Date of measurement	Body mass kg)	Mean (kg)	Log (body mass)
1	N024501	male	2017	0.240	0.227	−0.644
			2018	0.220		
			2019	0.220		
2	N024502	female	2017	0.250	0.247	−0.607
			2018	0.240		
			2019	0.250		
3	N024503	male	2018	0.240	0.235	−0.629
			2019	0.230		
4	N024504	male	2018	0.235	0.235	−0.629
5	N024507	female	2019	0.270	0.270	−0.569
6	N025509	female	2019	0.235	0.235	−0.629
7	Royal Palm	female	2017	0.275	0.275	−0.561
8	N024524	male	2019	0.240	0.240	−0.620
9	Thorvaldo	female	2017	0.270	0.270	−0.569
10	N024505	female	2018	0.270	0.253	−0.598
			2018	0.235		
11	N024506	female	2018	0.290	0.290	−0.538
