# Supplementary material for: A Comparison of Aerodynamic Parameters in Two Subspecies of the American Barn Owl (Tyto furcata)
Source: Animals (Basel). 2022 Sep 22;12(19):2532. doi: 10.3390/ani12192532 (PMC9558544; doi:10.3390/ani12192532)
Supplement: Supplementary file 1 [file animals-12-02532-s001.zip › animals-1900484-Table_S2_Johnson_Mikkola_data.pdf]

Table S2. Data from other studies used in Figure 4

Number	**Species**	**Author[1]**	**Log10 body mass (kg)[2]**	**Page[3]**	**Log10 wing loading (N/m^2)[4]**
1	Snowy Owl	Johnson	0.1414		1.7281
2	Great Horned Owl	Johnson	0.1285		1.7243
3	Eastern Screech Owl	Johnson	−0.7223		1.5964
4	Great Grey Owl	Johnson	0.0588		1.5599
5	Northern Pygmy-Owl	Johnson	−1.2295		1.5394
6	Barred Owl	Johnson	−0.1662		1.4959
7	Short-Eared Owl	Johnson	−0.4581		1.5102
8	American Barn Owl	Johnson	−0.3093		1.4968
9	Western Screech Owl	Johnson	−0.7501		1.4688
10	Northern Spotted Owl	Johnson	−0.2063		1.4571
11	Boreal Owl	Johnson	−0.8534		1.4450
12	Burrowing Owl	Johnson	−0.8368		1.4166
13	Long-Eared Owl	Johnson	−0.5841		1.3200
14	Flammulated Owl	Johnson	−1.2204		1.3097
15	Northern Saw-Whet Owl	Johnson	−1.0391		1.3458
16	Eagle Owl	Mikkola	0.4194	72	1.8429
17	Great Grey Owl	Mikkola	0.0128	179	1.5357
18	Ural Owl	Mikkola	−0.1824	163	1.5231
19	Tawny Owl	Mikkola	−0.2928	138	1.5937
20	Short-Eared Owl	Mikkola	−0.4196	234	1.5231
21	Long-Eared Owl	Mikkola	−0.5122	214	1.4830
22	European Barn Owl	Mikkola	−0.4685	39	1.4541
23	Tengmalm's Owl	Mikkola	−0.8386	257	1.4541
24	Pygmy Owl	Mikkola	−1.2076	113	1.4066

[1]References [14] (Johnson) and [29] (Mikkola)

[2]Data from column named "Weight" in Table 1 of [14] and from the different pages as specified in the next column from [29], mean values across males and females and across the different studies mentioned in the references

[3]page number in [29] from which body mass data were taken

[4]Data from column named "Wing-loading" in Table 1 of [14] (mean values across males and females and across the different studies) and from Table 2 in [29]
